# Mobile phone use and the welfare of community health nurses in Ghana: An analysis of unintended costs

**DOI:** 10.1016/j.wdp.2021.100317

**Published:** 2021-09

**Authors:** Albert Machistey Abane, Simon Mariwah, Samuel Asiedu Owusu, Adetayo Kasim, Elsbeth Robson, Kate Hampshire

**Affiliations:** a Department of Geography and Regional Planning, University of Cape Coast, Cape Coast, Ghana; b Directorate of Research, Innovation and Consultancy (DRIC), University of Cape Coast, Cape Coast, Ghana; c Durham Research Methods Centre, Durham University, United Kingdom; d Department of Geography, Environment and Earth Science, University of Hull, United Kingdom; e Department of Anthropology, Durham University, United Kingdom

**Keywords:** Mobile phones, Healthcare, mHealth, Community health nurses, Ghana

## Abstract

The use of mobile phones is fast transforming the healthcare delivery landscape in Ghana. A substantial number of health facilities are now dependent on mobile phones to facilitate their work. Evidence of the use of mobile phones in Ghana’s healthcare is however limited. In order to contribute to the evidence of the value of using mobile phones to promote healthcare, we interrogated and highlighted unexpected costs imposed on community health nurses who use their personal mobile phones for healthcare delivery in the country. Data for the study were derived from 598 completed questionnaires and extracts from focus group discussions with community health nurses who were sampled from three regions across the three main ecological zones of Ghana. The results show that over 90% of nurses bear the cost of paying for airtime, bundles and chargers used for work-related activities, yet less than 10% of them receive direct compensation. This costly burden has the potential to demotivate the nurses and threaten the country’s progress towards the achievement of universal health coverage. More significantly, the data strongly suggest that physical distance, regional location and gender are the main factors triggering extra costs incurred by the nurses. We conclude that the use of personal mobile phones for healthcare delivery imposed huge financial burden on community health workers in Ghana. A suggested intervention to forestall negative consequences on performance is to offer incentive packages to nurses as a compensation for the financial and non-physical costs of using personal mobile phones for work-related activities.

## Highlights

• The use of mobile phones are fast transforming health care delivery in Ghana.
• Community health nurses in Ghana use their personal mobile phones for work.
• However, over 90% of nurses bear the cost of using their mobile phones for work.
• This practice imposes huge financial burden on community health nurses in Ghana.
• The need for incentive packages to reduce the burden on nurses is imperative.

## 1 Introduction

A healthcare delivery system that seeks to achieve universal coverage (Ghana Health Service, 2017a, Ghana Health Service, 2017b) requires sufficient professional human capital (Abid et al., 2018; Mills et al., 2012; Opoku et al 2017). As a result, Ghana has, since the 1980s, been steadily increasing the provision of various health professionals. According to the Ghana Health Service, 2017b, Ghana Health Service, 2017a, in 2016 there were 3365 doctors, 14,791 community health nurses, 7662 midwives, 619 pharmacists, and 13,231 registered general nurses, with a doctor-population ratio of 1:84813. Though this ratio falls far below the WHO recommended ratio of 1:10,000, Ghana’s ratio is one of the best in sub-Saharan Africa (WHO, 2019). Currently, there are nearly 58,000 health workers deployed in various health facilities across the country (Ghana Health Service Annual Report, 2018). Meanwhile, a major challenge of delivery of health in the country is inadequate finance. Available data show that the country’s expenditure on health (5.2%), measured in terms of percentage of gross domestic product (GDP), is lower than the global average of 8.6% (World Health Organisation, 2015). However, the total general government expenditure on health (68.3%) is far higher than Africa’s average of 50.8% and the global average of 38.8% (World Health Organisation, 2015). In spite of these moderate strides, the country faces serious challenges when it comes to ensuring access to healthcare for all citizens. There are huge gaps between access for rural and urban dwellers and also between the rich and poor (Republic of Ghana, 2010, Ghana Health Service, 2015). There are also issues of health-worker dissatisfaction with conditions of service and the tools to work with, occasionally resulting in strikes.

As a result, at a Round Table Forum in 2015 to evaluate Ghana’s health situation in relation to the Millennium Development Goals, the Ministry of Health with support from UNDP pledged to increase efforts to meet the three goals directly related to health (*Goal 4 – Reduce child mortality; Goal 5 – Reduce maternal mortality; Goal 6 – Combat HIV/AIDS*, *malaria and other diseases*). The forum called for an up-scaling of effort across the country, which requires increasing the number of facilities and health professionals. In response, the Ghana Government built more hospitals at district and regional levels and established more community-based health planning and services (CHPS) compounds in rural settlements. Additional health professionals were also recruited to augment the existing staff (see Ministry of Finance, 2016 Budget Statement). These and other policy interventions ensured that community health nurses are deployed in several parts of the country, particularly rural communities. Generally, community health nurses in Ghana are trained licensed nurses whose core mandate is to provide preventive and curative maternal, newborn and child healthcare while residing in the communities they serve (MoH, 2016). Thus, Community Health Nurses (CHNs) are pivotal in assisting Ghana to meet the Sustainable Development Goal 3, target 3.2 - *reduce under-5 mortality to at least as low as 25 per 1000 live births by 2030;* and target 3.8 - *achieve universal health coverage including financial risk protection, access to quality essential healthcare services and access to safe, effective, quality and affordable essential medicines and vaccines for all by 2030*.

In this regard, Information, Communication and Technology (ICT) devices are transforming the healthcare landscape in many developing countries including Ghana. Mobile phones, in particular, have become conduits for interaction between healthcare workers and patients. Described as mHealth by the World Health Organisation (WHO, 2011) and others, these mobile telecommunication technologies are used in varied ways for healthcare delivery (e.g. to collect and process data from patients, follow up patients, provide appointment reminders), and patients including (seeking treatment advice, and accessing relevant information for accurate diagnosis of ailments) (Odendaal et al., 2019, Park, 2016, Akter and Ray, 2010, Aylward, 2011, Opoku et al., 2017, Watkins et al., 2018a). In effect, the face of healthcare delivery, especially in rural communities in developing countries where there is normally a shortage of health professionals, is rapidly changing with the emergence of these devices. However, some scholars and practitioners are skeptical about the effectiveness of mhealth innovations, with a few even suggesting that patients feel distracted and disregarded when health workers make references to their mobile phones in the course of consultation (Bloomfield et al., 2014; Watkins, Todd, Goudge, Gómez-Olivé, & Griffiths, 2018b). These reservations notwithstanding, the overall benefits of mHealth are generally believed to far outweigh the perceived concerns (Hampshire et al., 2015, Hampshire et al., 2017).

As a result, there is mounting evidence that some health personnel are adopting mobile phones and related devices to facilitate health delivery (Ghana Health Service, 2018, Hampshire et al., 2017, Kaplan, 2006, Okubeyejo and Eyesan, 2014). Indeed, mhealth innovations have become part of the established operations of some health facilities in Ghana. Some of the CHNs operating in these facilities are financially compensated when they formally use phones for work-related activities. However, a substantial number of others who also use their personal mobile phones to facilitate health delivery without official authorization are not compensated. They do so informally and, in spite of the substantial contribution to health delivery at the community level, very little has been done to recognize their efforts. In addition, very little research has focused on the activities of this category of CHNs in Ghana, the exceptions being two studies by Hampshire et al., 2017, Opoku et al., 2017. These studies in a limited manner, sought to assess the implications of using mobile phones to support health delivery and the possibility of up scaling.

During a pre-survey discussion, some of the CHNs equated their work to “a calling to serve”, however it is also the case that, like all other public workers, they have needs to be met but the salary and reward system does not cater for direct recompense for informal use of personal mobile phones for health delivery by CHNs. Ideally, all workers including CHNs should experience satisfaction in the work they do, because it is only a well-motivated, enthusiastic, passionate employee who remains committed to his/her organizational tasks (see Abid et al., 2018, Pehr, 2010). Therefore, the main objective of this paper was to measure and interrogate unintended financial and related psychological costs on CHNs using their personal mobile phones in delivering healthcare across Ghana. We tested the hypotheses that informal use of mobile phones to deliver healthcare has the potential to impose a significant financial burden on CHNs, with negative consequences on not only their welfare, but also perhaps on their motivation to engage positively with their work. It is our view that highlighting the nature and extent of the burden and related consequences on CHNs using their personal mobile phones in the course of their professional work can influence integration of these issues into future planning and policy for improved healthcare delivery in Ghana.

## 2 Contextual issues

Geographically, Ghana is a West African country with an estimated population of 30 million people, comprising a relatively young population − 38% below 15 years and 20% aged 15–24 years. Politically stable with an expanding economy in recent years, Ghana was rated by the World Bank as a lower middle-income in 2010 and the International Monetary Fund (IMF) describes the country as having bright economic prospects. However, like many African countries, Ghana is challenged in a number of areas, not least the health sector.

The history of primary healthcare in the country predates the Alma-Ata Declaration of 1978 which called for “Health-for-All” by the year 2000 (https://www.who.int/publications/almaata_declaration_en.pdf?ua=1). In 1977, Ghana introduced a variant of community health workers as Community Clinic Attendants and Traditional Birth Attendants (MoH 2016) to support health delivery in rural communities. This brought health services directly to people in their communities, and substantially reduced the referral of cases to secondary health facilities (Baatiema et al., 2016). However, lack of coordination and poor supervision led to a collapse of these groups of community health workers in the early 1990s (Baiden et al., 2007, Agyei-Baffour et al., 2012). The subsequent introduction of CHPS compounds gave birth to another cadre of trained health workers - Community Health Nurses licensed by the Nurses and Midwifery Council to operate in the CHPS compounds. A typical CHPS compound consists of an approved structure designed to deliver healthcare together with accommodation for CHNs (MoH, 2016). Health delivery at the CHPS compound is at the household level covering an area with a population of up to 5000 persons or 750 households where areas are densely populated. In addition to CHNs are Community Health Volunteers (CHVs) who are non-salaried members of the communities served by the CHPS compound. The CHVs are provided with special training to support the work of the CHWs. Their main functions include home visits to clients, weighing of children, assisting in family planning services, making referrals to Community Health Officers (CHOs) and mobilizing people for health education and environmental sanitation activities (MoH, 2016).

The introduction of mobile phones in formal healthcare delivery in Ghana was preceded by a number of pilots. One of the first was Mobile Technology for Community Health (MoTeCH), a partnership between Ghana Health Service (GHS), Grameen Foundation and Mailman School of Public Health of Columbia University in 2010. The programme had funding from the Bill and Melinda Gates Foundation and was piloted in the Upper East region of Ghana. The programme used low-cost java-enabled mobile phone technology to capture, transmit and process health service data collected by CHNs in their interactions with patients/clients (Grameen Foundation, 2012). For nurses with more sophisticated phones, they simply entered the information to transmit. Aside individual event data, the system also updated records on pregnancies, births, deaths, morbidities and health insurance status of clients. MoTeCH also introduced other mobile phone interrelated services such as the ‘Mobile Midwife’ application for alerts, reminders as well as information and advice to pregnant women and an application which allowed nurses to track the services rendered to women and newborns with opportunities for referrals (Foundation, 2011, Foundation, 2012). However, what is missing is the examination of the welfare of the staff providing mhealth care in both urban and rural communities, in particular, the financial and related costs incurred by informally using personal mobile phones and accessories to facilitate their work. Understanding the unintended costs of using personal mobile phones of CHNs will help address the Sustainable Development Goal 3, which seeks to ensure healthy lives and promote wellbeing for all at all ages (including CHNs).

## 3 Study methods

The paper relies on data from a much broader multi-country study on the informal use of mobile phones by community health nurses for health delivery in Ghana, Malawi and Ethiopia, known as the IMAGINE Project (Informal Mhealth in Africa: Grassroots Innovation and Networks), funded by Medical Research Council, UK. The study adopted both positivist and interpretive philosophies, and the sequential mixed-method approach, collecting empirical quantitative and qualitative data from CHNs. This methodology was informed by the main objective of the study, which sought to build an evidence base to support and enhance the informal use of mobile phones by CHNs for healthcare delivery. This paper draws only on the Ghana data, which were collected using survey (questionnaire) and focus group discussions (FGDs) with CHNs.

At the time the study commenced in 2018, Ghana had 10 regions; however, an additional six were created in the course of the study. Since the study was already far advanced before the other regions emerged in 2019, we decided to keep to the original 10 as the basis for the sampling of study sites. Thus, a three-stage sampling procedure was adopted: first, selection of a region from each of the three ecological zones of the country; second, selection of districts within the regions; and third, selection of the respondents from each district. Out of the 10 regions, the Central (coastal zone), Brong Ahafo (forest zone) and Northern (savannah zone) regions were randomly selected. The sample for the survey was by proportional to size, using a sampling weight of 0.346, 0.377 and 0.276 for Brong Ahafo, Central and the Northern regions, respectively based on the number of community health nurses in each region. Applied to the target sample size of 600 CHNs, the expected minimum number per region was 208 for Brong Ahafo, 226 for Central and 166 for the Northern region. In each region, five districts were randomly selected, to allow for adequate coverage of the sample size. In order to account for non-responses due to annual leave, casual leave, sick leave, and/or other contingencies, all community health nurses in the selected districts were surveyed (Table 1).

**Table 1:** Sampled Districts and CHNs for the Respective Regions.

Brong Ahafo Region	
Districts	Number of CHNs surveyed
Asunafo North	40
Dormaa East	38
Jaman North	38
Techiman Municipal	53
Wenchi Municipal	38
**Sub-Total**	**207**
**Central Region**	
Abura-Asebu-Kwamankese	41
Ajumako-Enyan-Essiam	65
Twifo Ati Mokwa	38
Upper Denkyira West	11
Ekumfi	34
**Sub-Total**	**189**
**Northern Region**	
Karaga	43
Sagnerigu	59
Sawla-Tuna-Kalba	19
West Gonja	42
West Manprusi	39
**Sub-Total**	**202**
**TOTAL**	**598**

Source: Field Survey, 2019.

The data collection exercise covered the period May 2018 to September 2019. This was preceded by the training of five field assistants, piloting of the data collection instruments and subsequent revision of the questionnaire based on observed field challenges. At the end of the data collection exercise, we obtained 598 usable completed questionnaires (with a response rate of 99.6%), comprising 207, 189, and 202 for Brong Ahafo, Central and Northern regions respectively.

In addition, qualitative data were collected through the use of FGDs to augment the findings from the survey data. In fact, the use of qualitative data enabled us to have a more informal, flexible conversation with research participants (Mariwah et al, 2019). Similarly, Hampshire et al. (2017) used qualitative approach to examine health-workers’ mobile phone practices and associated political-moral economies of care in Ghana and Malawi. In addition, Fletcher-Brown et al. (2020) used this approach to examine the acceptance of a knowledge-resource application by community health workers (CHWs) to deliver breast cancer healthcare in India. In our study, nine (9) FGDs, three (3) in each region, were conducted with CHNs, reflecting the different experiences in urban, *peri*-urban and rural areas across the regions. The FGDs were made up of 6–10 purposively selected participants, based on the availability of CHNs at the time of the data collection, and to account for their years of experiences as CHNs. The discussions were held at an agreed location, mostly in a health facility, and were facilitated by a trained research assistant and a note taker. Since each CHN could speak English, all the FGDs were conducted in the English Language.

The survey data was organized using SPSS version 21, and analysed using descriptive statistics and a multi-level linear regression. In order to map and examine the distance travelled by CHNs to offer services, geographic information system (GIS) tools were employed. On the other hand, the qualitative data were analysed manually based on the emerging themes. Thus, with expressed consent of the participants, the FGDs were audio-recorded and transcribed verbatim. The thematic analysis was done by developing a template that covered all the emerging themes from the study, such as accessibility to mobile phones, use of personal mobile phones for healthcare delivery and the costs associated with the use of personal mobile phones for work-related activities. This approach aided in comparing responses across the various respondents to provide rigorous qualitative insights (Mariwah, et al, 2021) into the unintended costs associated with the use of personal mobile phones for healthcare delivery.

The Ethical Review Committee of the Ghana Health Service, Ghana, and the Ethical Review Board of Durham University, UK provided ethical approval for this study.

## 4 Results

### 4.1 Socio-demographic characteristics of respondents

From the survey data, a typical community health nurse was found to be female (75%), aged 20–39 years (96%) and resident in rural settings (71%) (Table 2). He/she possessed the requisite professional qualification to practice. Indeed, about 83% (494) had professional nursing certificates while the rest either had diploma (16%) or degree (1%). The majority worked in CHPS compounds (54%) compared to health centres (28%), district hospitals (9%) or community/private hospitals (7%). Nearly two-thirds reported having worked for less than five years, with 30% of them having worked between five and nine years, and just two per cent over 14 years. Approximately 69% of them indicated earning monthly income ranging from GH¢1000 to GH¢1999 (equivalent GB£166.70 to GB£333.33) at the time of the survey.

**Table 2:** Brief background characteristics of community health nurses respondents.

Characteristics		Frequency	Per cent
Sex	Male	151	25.3
	Female	447	74.7
Age	20–29	296	49.5
	30–39	281	46.9
	40–49	11	1.8
	50–59	10	1.7
Education	Certificate	494	82.6
	Diploma	98	16.4
	Degree	6	1.0
Settlement type	Urban	172	28.8
	Rural	426	71.2
Health facility	District hospital	53	8.9
	Polyclinic	13	2.2
	Community/Rural hospital	42	7.0
	Health Centre	167	27.9
	CHPS compound	323	54.0
Income per month	<GH¢1000	168	28.1
	GH¢1000.00–1999.00	414	69.2
	GH¢2000.00–2999.00	15	2.5
	>GH¢3000.00	1	0.2

Source: Field survey, 2019.

### 4.2 Accessibility to health facilities

In order to visualize the physical space Community Health Nurses and their clients operate in, a map was constructed using geospatial data collected through a global positioning system (GPS) receiver in the field. The mapped points were plotted using the ArcPro software and projected from the geographic co-ordinates of World Geographic System WGS) 84 into the Ghana Metre Grid projected coordinate system. The processed data were interpolated using the distances patients have to travel to access the nearest health facility. Conversely, it demonstrates the distances and routes CHNs have to navigate in order to visit patients. The study used the inverse distance weighted (IDW) interpolation to model the farthest residence, based on the assumption that similar entities which are close to themselves by space are more related than those farther apart. In IDW, more weight is assigned to areas closer to the known point than spaces farther away. This is expressed as follows:

$$x^{*}=\frac{w_{1}x_{1}+w_{2}x_{2}+w_{3}x_{3}+\ldots +w_{n}x_{n}}{w_{1}+w_{2}+w_{3}+\ldots +w_{n}}$$

Where,

x* is the unknown value to be estimated from the known points;

x is the known point; and

w is the weight to be assigned.

**Fig. 1:**
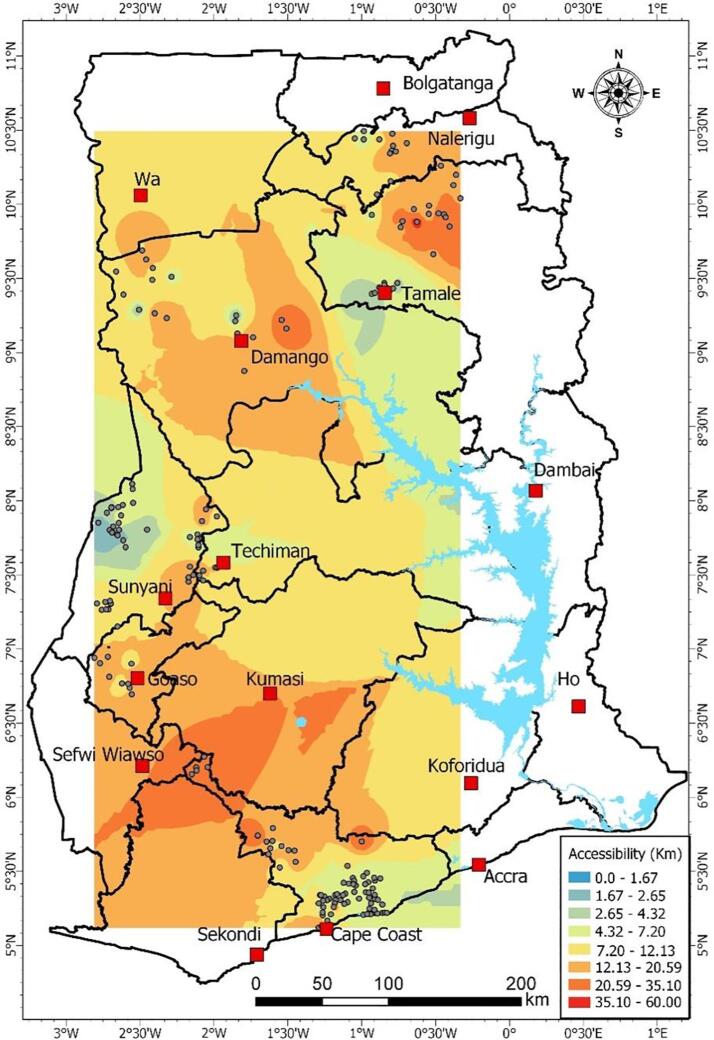
Spatial distance patients journey to access a health. Source: Fieldwork, 2019.

The weight assigned is the inverse distance of a point to each known point value. To reduce the level of error with the estimation, the model was undertaken for only the geographic location of the mapped health facilities rather than extrapolate for the entire country of Ghana. As shown in Fig. 1, the majority of the patients were likely to travel less than a kilometer (km) to access healthcare in the three study regions. However, the mean distance patients could likely cover was slightly more than 11 km. Within the coastal zone, especially around Cape Coast Metropolis, access to health facilities in terms of distance was estimated at a range of 4.32–7.20 km. Moving inland, accessibility reduced substantially with distances exceeding 20 km for Dunkwa and Diaso. In the Brong Ahafo region, Goaso area had the lowest levels of accessibility, returning estimated distances of less than 3 km. In the northern half of the country, Tamale recorded the shortest distances to health facilities, ranging between 1.67 and 2.65 km. The remaining communities had values exceeding 20 km.

Given the scenario where either CHNs or patients have to travel long distances to access or deliver healthcare, there is a high probability that some of them would rely on their mobile phones to accomplish tasks that do not necessarily require a physical presence.

### 4.3 Landlines, mobile phones and healthcare delivery

The delivery of mHealth depends on availability of ICT technologies including mobile phones and landlines. Across all the health facilities included in the study, only about 7% (41) of the respondents reported having fixed telephone lines (i.e. landlines). In terms of workplace mobile phones, only 107 (18%) out of the 598 CHNs responded in the affirmative. However, nearly all (99.7%) of the community health nurses said they owned mobile phones, 92% of them internet-enabled, which they used every day/most days (90.5%) to facilitate their work, with slight variations across the three regions (Table 3). Subjected to Pearson’s Chi –square test, the results across regions showed significant differences in the three regions (X^2^ = 12.498p value = 0.014) in the use of mobile phones for work.

**Table 3:** Regularity of use of personal mobile phone for work per week by region.

Regularity	Region		
	Brong Ahafo	Central	Northern
	Freq. %	Freq. %	Freq. %
Every day/Most days	187 90.8	162 85.7	192 95.0
At least once a week	19 9.2	24 12.7	9 4.5
At least once a month	0 0.0	3 1.6	1 0.5
**Total**	**206 100**	**189 100**	**202 100**
**Per cent of Total**	**34.5**	**31.7**	**33.8**

Source: Field survey, 2019.

### 4.4 Work-related mobile phone use

Once it was clear that a substantial number of CHNs use their own mobile phones to facilitate their activities, we then sought to establish exactly the services where their personal phones became necessary. As indicated in Table 4, the areas were many and varied including voice calls (98.7%), messages to patients, colleagues and volunteers (71.7%); participating in WhatsApp group (77.9%), using Google search to obtain information ((74.7%) and taking pictures or videos of health-related events or activities (73.7%).

**Table 4:** Unofficial application of mobile phones for Informal Mhealth.

Unofficial mobile phone application	Reported in the Preceding 4 Weeks			
	Yes		No	
	N	%	N	%
Voice calls	589	98.7	8	1.3
SMS (to communicate with patients, colleagues, volunteers, etc.)	428	71.7	169	28.3
SMS to send reports, data or information	250	41.9	347	58.1
WhatsApp (or similar) to send reports, data or information	365	61.1	232	38.9
WhatsApp (or similar) to contact someone (e.g. patient, colleague)	376	63.0	221	37.0
Participating in a WhatsApp Group (e.g. of fellow health-workers)	465	77.9	132	22.1
Establishing a WhatsApp Group (e.g. of fellow health-workers)	166	27.8	431	72.2
Facebook or other networking site to seek information	107	17.9	490	81.9
Google (or other internet search) to get information	446	74.7	151	25.3
Downloaded health-related Apps (e.g. from Google playstore)	205	34.3	392	65.7
Notepad (or similar) for making notes	48	8.0	549	92.0
Camera/video: Taking pictures/film of activities /events	440	73.7	157	26.3
Camera: Taking shots of reports / paperwork	411	68.8	186	31.2
Camera: Taking pictures of patient symptoms (to seek advice)	330	55.3	267	44.7
Voice recording (e.g. for recording meetings or any other purpose)	72	12.1	525	87.9
Calculator: for collecting data or making reports	531	88.9	6	11.1
Calculator: calculating medicine dosages	462	77.4	135	22.6
Torch to work in the night	355	59.5	242	40.5
Torch for patient examination	209	35.0	388	65.0
Stopwatch (e.g. for taking pulse or breathing rate	210	35.2	387	64.8
Mobile money to collect/send payments or allowances	111	18.6	487	81.4
Others (Calendar, emails)	272	45.6	325	54.4

Source: Fieldwork, 2019.

The nature of the work therefore makes it necessary to contact other experts for either advice or referrals. As one 33 year old female nurse in a regional hospital remarked: *I am in the family planning unit and … day in and out we call … more than 5 minutes and it’s your own credit* … *you call other people, other doctors, other workers to come in to help.* Another 31 year-old CHN expressed how she uses her mobile phone to send health reports, which of course, comes with the cost of internet data: *Sometimes you may be contacted by any of these superiors or colleagues for a report. All you need to do is to use your phone to take a snapshot of the report and send it via WhatsApp to whoever requested for it.*

### 4.5 Estimated costs of using personal mobile phones to facilitate work

The use of mobile phones for healthcare delivery has cost (financial) implications for CHNs. The data showed that a substantial proportion of the CHNs who used their own mobile phones for work-related activities incurred significant financial costs relating to purchase of airtime, data bundles, chargers and electricity (See Table 5).The following excerpts from the FGDs further reveal some of the financial burden imposed by the use of personal mobile phones for healthcare delivery:

It is a problem; you will buy credit GHS 10 and within 3 days because of calling the clients, it will be finished. [31-year old female CHN, n Northern region]

It has been a cost to us because we have to use our money to buy call credits and data and use it for the purposes of our work…I am unable to tell precisely, but if for instance I buy GHS 10 worth of credit, about 70% of it is spent on my work as a nurse. [27-year old male CHN,Brong Ahafo region]

I must confess that those colleagues at the very remote areas call a lot. For instance, our colleagues at Timber Nkwanta (Timber Junction) call every single day for one reason or the other – requesting for this and that. This seriously is a drain to them. Aside that, if you miss any of their calls, you will have to return all those calls, and that becomes a cost to us as well. Again, even though we may receive a letter or note when someone is referred, most of the time, the receiving facility may require further explanation, and that means calling. Seriously, sometimes you may have to spend close to 3 or 4 hours trying to get to the bottom of the case [32-year old male CHN, Brong Ahafo region]

**Table 5:** Expenditure on phone for work-related activities.

Amount per week	Frequency	Percent
Less than 5 GHS	193	32.3
GHS 5–10	346	58.0
More than GHS 10	58	9.7
Total	597	100.0

Source: Field survey, 2019.

N/B: At the time of data collection, GB£1.00 = GHS 6.00.

In addition, we estimated the costs (in British Pounds Sterling [£]) of airtime and data bundles using a generalized linear regression model with Gaussian family and log-link to account for any skewed distribution normally associated with cost data (Table 6). Logarithm transformation of the data before analysis was not appropriate because the data contained true zero values.

**Table 6:** . Estimation of extra cost of informal mobile health delivery on CHNs in Ghana.

Variable	Airtime Cost Log (£)		Data Cost Log (£)		Total Cost Log(£)		Time Cost Log (min/day)	
	Est(95% CI)	P value	Est(95% CI)	P value	Est(95% CI)	P value	Est(95% CI)	P value
Intercept	−0.18 (-0.45,0.06)	0.1676	0.00 (-0.36, 0.34)	0.9849	0.60 (0.31, 0.88)	0.0000	4.48 (4.12, 4.81)	0.0000
Allowance (Ref = Yes)	0.19 (-0.21, 0.49)	0.2714	−0.42 (-2.01, 0.19)	0.3103	−0.15 (-0.83, 0.28)	0.5713	0.05 (-0.54, 0.45)	0.8100
Gender (Ref = M)	−0.22 (-0.36, −0.07)	0.0032*	−0.09 (-0.29, 0.12)	0.3767	−0.13 (-0.30, 0.04)	0.1102	−0.15 (-0.35, 0.06)	0.1453
Rural (Ref = urban)	0.08 (-0.08, 0.24)	0.3424	−0.02 (-0.22, 0.20)	0.8442	−0.00 (-0.17, 0.17)	0.9625	−0.04 (-0.25, 0.19)	0.7191
Emp. Duration	0.00 (0.00, 0.03)	0.0308*	0.01 (-0.01, 0.03)	0.4263	0.01 (-0.00, 0.03)	0.1380	0.00 (-0.02, 0.02)	0.8334
Distance	0.00 (0.00, 0.1)	0.2468	0.00 (-0.01, 0.01)	0.9275	0.00 (-0.01, 0.01)	0.6529	0.00 (-0.00, 0.01)	0.2246
Region (Ref = Central)								
Brong Ahafo	0.20 (0.03, 0.38)	0.0275*	0.29 (0.04, 0.50)	0.0251*	0.24 (0.05, 0.44)	0.0142*	0.12 (-0.09, 0.34)	0.2703
Northern	0.16 (-0.02, 0.35)	0.0784	0.05 (-0.21, 0.3)	0.7087	0.12 (-0.08, 0.34)	0.2401	−0.16 (-0.43, 0.10)	0.1971

Source: Field survey, 2019.

For the analytical model used, let $Y_{\mathrm{ij}}$be the cost of airtme by a community health nurse i from districtj, the generalised linear model was formulated as $Y_{\mathrm{ij}}\;N\left(\mu_{\mathrm{ij}},\sigma^{2}\right)$, where

$$\mu_{\mathrm{ij}})=\beta_{0}+\beta_{1}Allowance+\beta_{2}Gender+\beta_{3}Year+\beta_{4}Dist+\beta_{5}Dist+\beta_{6}BR+\beta_{7}North$$

It is important to note that $\sigma^{2}$ is constant for community health nurses because of the lack of heterogeneity between the districts. The average difference in cost of airtime was captured by $\beta_{1}$, where a positive value means that those receiving airtime allowances spent more than those not receiving airtime allowances. The difference in cost between female and male community nurses was captured by $\beta_{2}$ where a positive value implies that female community health nurses spent more on airtime than males. $\beta_{3}$ was used to represent the gradient between cost of airtime and the year of employment as a community health nurse. A positive value means that the amount spent on airtime increases with year of experience. Similarly, a positive value for $\beta_{4}$ means community health nurses in catchment areas farther from their health centres spent more on airtime. A positive value for $\beta_{5}$ means that those in rural areas spent more on mobile phone calls than those in urban areas. Lastly, the model also investigates whether the amount spent on airtime differs between regions. A positive value for $\beta_{6}$ is an indication that CHNs in the Brong Ahafo region spent more on airtime and data bundles than their counterparts from the Central region. Similarly, a positive value for $\beta_{7}$ suggests that CHNs in the Northern region spent more on airtime than their colleagues in the Central region. Following the same principle, other outcome data were analysed using generalised linear models.

As observed in Table 6, three variables emerged as significantly influencing additional costs incurred by CHNs using their phones to deliver healthcare: gender, employment duration and regional location. Male community health nurses tend to attract significant additional costs on airtime (p = 0.0032) compared to their female counterparts. The results also suggest that incurring additional costs on airtime depends largely on how long CHNs have been in their job. CHNs with over six years of work experience tend to spend far more than those who have been on the job for less than six years; probably due to the fact that they may have built larger network of clients and colleagues. As was observed in earlier cases, the propensity of CHNs attracting additional costs on both airtime and data bundles also depends on the distance to where their clients live. To reduce long trips to reach clients, CHNs prefer to make calls. Also, the CHNs working in the Brong Ahafo region generally spend more on the use of their personal mobile phones for health delivery than those in the Central and Northern regions. There was no significant difference in additional costs CHNs incurred between the Northern and Central regions.

## 5 Discussion

The study has brought to the fore issues burdening CHNs that require further interrogation. First, the CHNs in this study were mainly young women who generally operated at CHPS compounds. This reinforces the prevailing perception that the health professions, especially in supporting staff roles, is dominated by females (Ghana Health Service, 2017a, Ghana Health Service, 2017b).

Second, the spatial distribution of health facilities where CHNs are based showed that Ghana is still challenged spatially when it comes to comparative access to health services between rural and urban locales. The majority of urban residents have better proximity to health facilities compared to populations in rural areas where health facilities are generally limited, in spite of Government policy to bridge the rural–urban divide (MoH, 2017). It is clear from our study that patients from some rural communities had to traverse long distances to reach health facilities. Conversely, rural-based community health nurses also face such challenges in order to reach their clients.

Third, the results strongly suggested that spatial differences between the location of health facilities and clients’ residences play an important role in CHNs having to make calls as part of their work. Rather than making long trips to reach their clients, the nurses find solace in relying on their mobile phones to bridge the distance gap. In doing so, however, the CHNs incur significant extra costs relating to airtime and data bundles, which are not reimbursed for reasons that they are unauthorized and only at the discretion of the CHNs themselves.

In the literature, there are many examples of mobile phones being used to improve delivery of healthcare services. For example, Watkins et al. (2018a) employed text messages to patients to remind them to take their medications and also confirm their appointments. In Uganda, an NGO called ‘Text to Change’ collaborated with a telecom company to increase awareness of HIV/AIDS among young people as well as encourage them to seek HIV testing and treatment (see Watkins et al., 2018b). The difference between these examples and that reported in this paper is that the community health nurses in Ghana are using their own mobile phones to informally facilitate their professional work and bear the associated financial costs. Thus, a substantial number of health workers, particularly the CHNs, use their own mobile phones to facilitate components of their work. This was found to be fairly widespread across the three study regions and demonstrates the capacity of the CHNs to use ICT (specifically mobile phones and related applications) to improve efficiency of health service delivery (see also Hampshire et al., 2017, Kaplan, 2006, Okubeyejo and Eyesan, 2014). A 32-year old male CHN working in a *peri*-urban settlement in the Savannah zone had this to say when asked why he used his own airtime and data bundles to perform official duties: *You cannot be in the system without saying that you don’t want to use your own credit to call somebody. It is part of our work … you know that you are there to save lives so whatever you will do to save lives, you don’t care about that* (i. e. cost of airtime and data bundle).

Fifth, it was found that a few CHNs received monetary compensation for using their own mobile phones to make calls in the course of their work. Some of the nurses described the allowances paid to them as grossly inadequate. However, for the majority who remained unrewarded for the extra costs incurred, they had to carry huge burdens as the statistical model showed, especially for those serving in CHPS compounds in rural areas in the Brong Ahafo and Northern regions. There is sufficient reason to suggest that over time, the costs could potentially demotivate them and reduce their productivity if nothing is done to reverse the trend. Although a few of them claimed that *they see their job as a calling to service because you are there to save lives* (32 year old male CHN) (see also (Watkins et al., 2018b, Watkins et al., 2018a), others were happy to call for extra support by way of incentives. *As for the credit when we get it, it will really help but GHS 20 won’t be enough… may be about GHS 40 or 50 wouldn’t be bad for a month* (26 year old female CHN in charge of CHPS compound)*.* One respondent was even more emphatic in her request: *even GHS 100 will not be enough, because* (the calls are many), *day in day out and we do referrals* (30 year old female CHN).

Sixth, in the literature, some researchers have proposed non-financial compensation for workers facing challenges similar to those of the CHNs in this study. They recommend giving the workers a day or two off in lieu of monetary rewards or an opportunity for further training (Uwakweh, 2012, Bhatt et al., 2008), while they also caution that it could lead to discrimination with consequences of worker apathy if this becomes a permanent feature. From the perspectives of many of the CHNs in this study, monetary rewards would be more appreciated than non-physical benefits (see also Hampshire et al., 2017). As a 30-year old CHN working in a rural CHPS compound in the Northern region remarked, *I think we are working for Ghana, so I think the government should add something small to our salary to be used for call credit.* Put into context, the request is understandable because of the low levels of income and pervasive poverty reported among residents in the rural and *peri*-urban communities in the country, especially the Northern and Central regions which are among the poorest in the country (Ghana Statistical Service (GSS), Ghana Health Service (GHS), and ICF International, 2015). Monetary rewards would enable the CHNs to cope with expected daily commitments as well as defray the expenses incurred on airtime, data bundles, electricity and repairing their mobile phones.

## 6 Conclusion

In conclusion, the paper has demonstrated that CHNs in Ghana incur significant financial costs in using their personal mobile phones for work-related activities. Such costs do not only drain their salaries, but may also serve as a demotivation to continue using their personal phones to facilitate healthcare delivery. For now, some of the CHNs describe their job as a “calling to serve humanity” and that this may urge them to continue to spend money on credits for health related-activities. However, we cannot forever rely on their good will and benevolence, because this may constitute *cost shifting* where health systems inadvertently exploit the moral imperative to care by lower-level health workers such as CHNs who are among the least paid (Hampshire et al., 2017).

Therefore, we recommend that the Ghana Health Service should liaise with its relevant ministry and development partners to critically review the activities of CHNs with a focus on the expenses incurred by them when they use their own mobile phones to deliver healthcare. A scheme should be devised to provide some form of compensation for them to defray the extra costs incurred and also motivate them to continue to improve health delivery through mHealth. In this regard, the Ministry of Health can negotiate with telecommunication companies to provide some phone credits to CHNs as part of their corporate social responsibility. Furthermore, efforts could be made to begin a national discussion on how to support mHealth to expand without necessarily imposing additional costs on the CHNs. Achieving such as useful balance may enhance healthcare delivery and contribute to the achievement of universal health coverage in Ghana.

Though the study provides a useful case study on the unintended costs imposed by the use of personal mobile phones for healthcare delivery in Ghana, we recommend that future research should explore approaches to engage relevant stakeholders in order to achieve a balance in scaling up informal mhealth devoid of *cost shifting* to vulnerable heath workers.

## Funding

This research project was funded by the Medical Research Council (MRC), UK (Grant Ref: MR/R003963/1)

## Declaration of Competing Interest

The authors declare that they have no known competing financial interests or personal relationships that could have appeared to influence the work reported in this paper.
